# A novel household‐based patient outreach pilot program to boost late‐season influenza vaccination rates during the COVID‐19 pandemic

**DOI:** 10.1111/irv.13041

**Published:** 2022-09-13

**Authors:** Lloyd Fisher, Matthew M. Loiacono, Nick Payne, Tina Kelley, Michael Greenberg, Mary Charpentier, Candace Leblanc, Devi Sundaresan, Tim Bancroft, Andrea Steffens, Misti Paudel

**Affiliations:** ^1^ Reliant Medical Group Worcester Massachusetts USA; ^2^ UMass Medical School Worcester Massachusetts USA; ^3^ Sanofi Swiftwater Pennsylvania USA; ^4^ Optum Life Sciences Eden Prairie Minnesota USA

**Keywords:** influenza, late season, vaccination

## Abstract

**Background:**

The objective of this study was to test a novel household‐based approach to improve late‐season influenza vaccine uptake during the 2020–2021 season, using Epic's MyChart patient portal messages and/or interactive voice response telephone calls.

**Methods:**

This study was a non‐blinded, quality improvement program using a block randomized design conducted among patients from Reliant Medical Group clinics residing in a traditional household (≥2 individuals clinically active in the Reliant system living at the same address). Households were randomized 1:1:1 into intervention arms: non‐tailored communication (messaging based on CDC's seasonal influenza vaccination campaign), tailored communication (comprehensive communication including reinforcement of the importance of influenza vaccination for high‐risk individuals), and standard‐of‐care control. Influenza vaccination during the program was captured via medical records, and the odds of vaccination among communication arms versus the control arm were assessed. A survey assessing influenza vaccination drivers was administered using MyChart.

**Results:**

Influenza vaccination increased by 3.3% during the program period, and no significant differences in vaccination were observed in intervention arms relative to the control arm. Study operationalization faced substantial challenges related to the concurrent COVID‐19 pandemic. Compared with vaccinated survey respondents, unvaccinated respondents less frequently reported receiving a recommendation for influenza vaccination from their healthcare provider (15.8% vs. 42.3%, *p* < 0.001) or awareness that vaccination could protect themselves and higher risk contacts (82.3% vs. 92.6%, *p* < 0.001).

**Conclusions:**

No significant effects of the interventions were observed. Survey results highlighted the importance of healthcare provider recommendations and the need for increased education around the benefits of vaccination.

## 1 INTRODUCTION

During the 2019–2020 season, the CDC estimated 35 million cases, 380,000 hospitalizations, and 20,000 deaths were attributable to influenza in the United States, with an annual economic burden of $11.2 billion.^1^, ^2^ Severe influenza complications, including hospitalizations and death, disproportionately affect individuals ≥65 years of age; however, pregnant persons, young children, and those with chronic health conditions and disabilities are also at a higher risk for severe influenza‐related illness than younger healthier adults.

Influenza vaccination is the most effective method to prevent influenza infections and associated hospitalizations and deaths. During the 2019–2020 season, an estimated 7.5 million cases, 105,000 influenza‐related hospitalizations, and 6300 influenza‐related deaths were averted due to influenza vaccination in the United States.^3^ Despite a multitude of efforts to improve the uptake of seasonal influenza vaccines,^4^, ^5^, ^6^, ^7^ rates have remained stagnant in the United States over the past 10 years.^8^ While rates among children and individuals >65 years have come close to the national target of 70%, those among younger adults have consistently lagged behind.^9^

In the wake of the COVID‐19 pandemic, public health experts urged the importance of influenza vaccination to mitigate the increased burden of the “twindemic” to the population and healthcare system. The COVID‐19 pandemic also marked a turning point in the perception of public health, with an increased emphasis on protection of those around you and thinking in terms of units (i.e., household and family), with regard to virus transmission. This unique situation highlights the importance of both addressing stagnant influenza vaccination rates and ensuring that vaccine uptake is sustained throughout the latter part of the season, a time when uptake traditionally slows, yet influenza can still pose a substantial risk.^10^, ^11^

Leveraging the public's recently heightened awareness of infectious disease and vaccination, we sought to explore a novel household‐based approach to improve late‐season influenza vaccine uptake during the 2020–2021 season, using messages from the Epic (Epic Systems Corporation, Verona, WI) MyChart patient portal (MyChart) and/or interactive voice response (IVR) telephone calls. By targeting all members within a household, regardless of their influenza vaccination status at the time of outreach, we aimed to encourage positive dialogue around influenza vaccination among household members and reinforce the shared responsibility of vaccination to protect oneself and those around you.

## 2 METHODS

### 2.1 Study design and population

This study was a non‐blinded, quality improvement program using a block randomized design conducted among patients from Reliant Medical Group clinics (Reliant) in central Massachusetts. To be eligible for program participation, individuals must have been ≥6 months of age at the time of randomization and clinically active in the Reliant system (i.e., ≥1 clinic visit at a participating Reliant clinic in the past 36 months if aged <65 years, or in the past 18 months if aged ≥65 years). Individuals were excluded from the program if they were unable to receive an influenza vaccination (i.e., history of allergic reaction or adverse event resulting from an influenza vaccination, history of Guillain–Barre syndrome) or did not reside in a traditional household (e.g., residents of nursing homes, institutions, long‐term care facilities, or group settings [e.g., boarding school or dormitory]). Additional inclusion criteria were assigned based on household status. Households were defined as ≥2 individuals clinically active in the Reliant system who resided at the same household address. The household must have had ≥1 member who was ≥18 years of age at the time of randomization or had a proxy MyChart account or phone number for a parent or guardian, met the inclusion criteria and was not yet vaccinated at the time of randomization, had not opted out of communications or was not on the do‐not‐call list, and had an active electronic health record (EHR) portal status and a valid phone number.

### 2.2 Program cohorts and randomization

**FIGURE 1:**
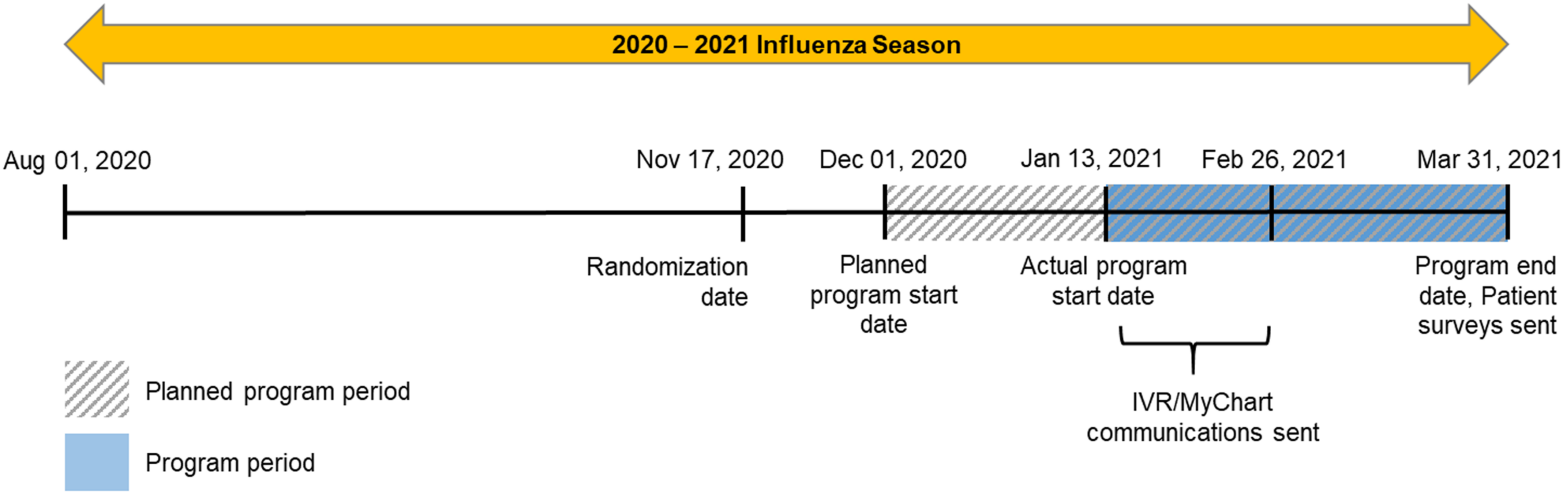
Study timeline

Eligible households were stratified based on risk, which included a high‐risk cohort (≥1 household member with a risk factor for severe influenza‐ or COVID‐19‐related complications) and a non‐high‐risk cohort (no household members with known risk factors). High‐risk conditions were based on current problem lists (Table S1). Age (i.e., <2 or ≥65 years) alone was not considered as a risk factor in this stratification. Eligible households were randomized 1:1:1 between the control, non‐tailored, and tailored communication arms, blocking on clinic/region and household high‐risk status. Blocking on risk status ensured high‐risk and non‐high‐risk households were balanced across study arms. Individuals in the control arm received no additional communication outside of Reliant's standard‐of‐care (SoC) communications (e.g., an influenza vaccination reminder email sent September 2020 and occasional social media postings advertising vaccination clinics through October 2020). The non‐tailored communication arm received MyChart or IVR messaging based on CDC's seasonal influenza vaccination campaign materials.^12^ The tailored communication arm received a more comprehensive version of the non‐tailored communication, including additional information reinforcing the importance of influenza vaccination for high‐risk individuals. Full contents of the non‐tailored and tailored communications are provided in Table S2. Randomization was performed by Reliant on November 17, 2020 for all eligible households (Figure 1).

### 2.3 Program period

The program period was defined as the period from the initiation of household communication through the end of follow‐up and was scheduled to begin on December 1, 2020 (Figure 1). Due to pandemic‐related operational challenges, the start of the program period was delayed until January 13, 2021. Individuals were followed through March 31, 2021 to ascertain influenza vaccination status.

### 2.4 Communications

Non‐tailored and tailored communications were sent to eligible household members via MyChart or IVR beginning January 13, 2021 and were delivered on a rolling basis through February 26, 2021 (Figure 1). Both communications urged individuals to call and schedule a household vaccination appointment at their clinic and/or provided a link (MyChart) to directly schedule appointments.

### 2.5 Participant survey

To determine the factors that influenced program participants' decision on whether to get vaccinated or not, a survey was administered to all active MyChart participants. Completed surveys were linked to the participant's EHR using an encrypted identifier.

### 2.6 Ethics approval and patient consent

Prior to data collection, the study was approved by the WCG Institutional Review Board (NEIRB #1295416; October 30, 2020) and a waiver of patient consent was granted under 45 CFR 46116(f). All communications and survey materials were additionally reviewed and approved by Reliant Medical Group's Communications Office.

### 2.7 Study measures

Household demographics, participant demographic and clinical characteristics, and vaccination rates (based on individual‐level EHR‐recorded influenza vaccinations) were presented for the eligible program population. Demographics and survey results were presented for those who returned a completed survey.

### 2.8 Analysis

Household characteristics and patient demographic and clinical characteristics were assessed descriptively. To assess randomization balance across tailored, non‐tailored, and control arms, household characteristics were compared using Pearson's chi‐square test. Patient characteristics were compared using the Rao–Scott test to account for correlation among patients from the same household. Among the subset of individuals not yet vaccinated at program start, logistic regression was used to model the unadjusted odds of vaccination during the program period among non‐tailored and tailored communication arms, relative to the SoC control arm. Odds ratios and 95% confidence intervals were estimated. As a result of COVID‐19‐related logistical issues, not all patients who were set to receive IVR communications were contacted (*n* = 11,698 patients [34.1%]). To assess the impact of early termination of IVR communication, a sensitivity analysis was conducted where logistic regression was used to model the unadjusted odds of vaccination during the program period among control households and tailored/non‐tailored households where ≥1 member received a MyChart or IVR communication. Descriptive statistics are presented for the full randomized sample, but results are otherwise focused on the subset of eligible households (i.e., ≥1 household member unvaccinated) as of the delayed program start. Participant survey responses were compared between vaccinated and unvaccinated respondents using the Rao–Scott test.

## 3 RESULTS

### 3.1 Randomization and program population characteristics

**FIGURE 2:**
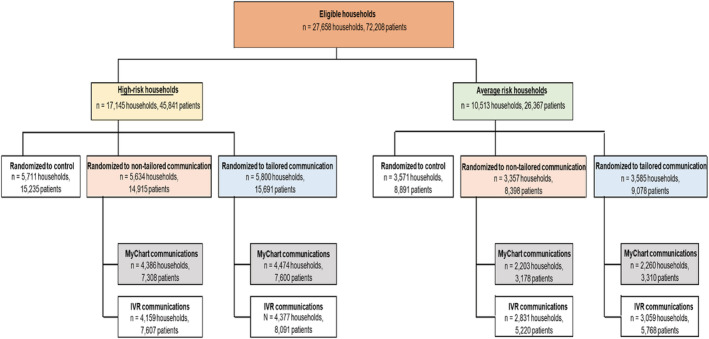
Randomization scheme of the eligible study population. IVR, interactive voice response

A total of 36,920 households (94,747 individuals) were initially randomized (Figure S1). The proportion of households included in the initial randomization from each Reliant clinic was balanced across program arms (Table S3). Given the delay in the program start date, the number of eligible households (i.e., ≥ 1 household member unvaccinated at program start) decreased to 27,658 households (72,208 individuals) (Figure 2). After excluding ineligible households, the balance between program arms and across clinics remained comparable to that of the initially randomized population (Table S3).

Among these eligible households, most individuals were female (52.7%), aged 18–49 years (43.7%), White (63.9%), had commercial insurance (64.6%), and had an active MyChart status (65.0%) (Table 1). Most patients were considered non‐high‐risk (65.0%) and 33.3% were vaccinated prior to the start of the intervention program. There were statistically significant differences among the intervention cohorts by health plan type and race/ethnicity, albeit of small magnitudes.

**TABLE 1:** Demographic characteristics of individuals in eligible households

	Total (*n* = 72,208)	Control (*n* = 24,126)	Non‐tailored communication (*n* = 23,313)	Tailored communication (*n* = 24,769)	*p* value
Age group, *n* (%)					0.058
<18 years	20,006 (27.7)	6655 (27.6)	6389 (27.4)	6962 (28.1)	
18–49 years	31,521 (43.7)	10,542 (43.7)	10,174 (43.6)	10,805 (43.6)	
50–64 years	14,684 (20.3)	4978 (20.6)	4687 (20.1)	5019 (20.3)	
≥65 years	5997 (8.3)	1951 (8.1)	2063 (8.9)	1983 (8.0)	
Sex, *n* (%)					0.921
Female	38,050 (52.7)	12,706 (52.7)	12,312 (52.8)	13,032 (52.6)	
Male	34,154 (47.3)	11,419 (47.3)	10,999 (47.2)	11,736 (47.4)	
Nonbinary/missing	4 (0.01)	1 (0.0)	2 (0.01)	1 (0.0)	
Race/ethnicity, *n* (%)					0.001
White	46,167 (63.9)	15,269 (63.3)	15,082 (64.7)	15,816 (63.9)	
Black	2622 (3.6)	886 (3.7)	836 (3.6)	900 (3.6)	
Hispanic	4171 (5.8)	1370 (5.7)	1313 (5.6)	1488 (6.0)	
Asian/Pacific Islander	3943 (5.5)	1500 (6.2)	1148 (4.9)	1295 (5.2)	
Native American	219 (0.3)	79 (0.3)	69 (0.3)	71 (0.3)	
Unknown	14,414 (20.0)	4802 (19.9)	4673 (20.0)	4939 (19.9)	
Missing	672 (0.9)	220 (0.9)	192 (0.8)	260 (1.1)	
MyChart active status, *n* (%)	46,932 (65.0)	15,660 (64.9)	15,235 (65.4)	16,037 (64.8)	0.525
Health plan type, *n* (%)					0.011
Commercial	46,653 (64.6)	15,680 (65.0)	15,044 (64.5)	15,929 (64.3)	
Medicare	5343 (7.4)	1741 (7.2)	1845 (7.9)	1757 (7.1)	
Medicaid	12,145 (16.8)	3960 (16.4)	3826 (16.4)	4359 (17.6)	
Medicare/Medicaid dual eligible	83 (0.1)	28 (0.1)	32 (0.1)	23 (0.1)	
Other/unknown	7984 (11.1)	2717 (11.3)	2566 (11.0)	2701 (10.9)	
Influenza risk status, *n* (%)					0.100
Average risk	46,908 (65.0)	15,778 (65.4)	15,005 (64.4)	16,125 (65.1)	
High risk	25,300 (35.0)	8348 (34.6)	8308 (35.6)	8644 (34.9)	
Vaccinated prior to program start (8/1/2020 to 1/12/2021)^a^					0.370
Yes	24,025 (33.3)	8053 (33.4)	7816 (33.5)	8156 (32.9)	
No	48,183 (66.7)	16,073 (66.6)	15,497 (66.5)	16,613 (67.1)	
Vaccinated during influenza season (8/1/2020 to 3/31/2021)^a^					0.565
Yes	26,414 (36.6)	8834 (36.6)	8583 (36.8)	8997 (36.3)	
No	45,794 (63.4)	15,292 (63.4)	14,730 (63.2)	15,772 (63.7)	

*Note*: Excludes households where all members were fully vaccinated prior to the program start (08/01/2020–1/12/2021).

^a^ Based on data from electronic health records.

### 3.2 Vaccination rates

Among individuals initially randomized, 51.7% of patients were vaccinated during the influenza season (8/1/2020–3/31/2021), of which, 49.2% were vaccinated prior to the start of the program (Table S4). Among individuals in eligible households (i.e., those in which ≥1 member remained unvaccinated for influenza at the start of the program), 33.4%, 33.5%, and 32.9% in the control, non‐tailored communication, and tailored communication intervention arms (*p* = 0.370) were fully vaccinated at program start (Table 1). At the end of the program period, an additional 3.3% of individuals (*n* = 2389) in eligible households were vaccinated. By program arm, individual vaccination rates were 36.6%, 36.8%, and 36.3% in the control, non‐tailored communication, and tailored communication intervention arms, respectively (*p* = 0.565). The odds of receiving an influenza vaccine during the program period were 1.02 (95% CI = 0.91, 1.14) for individuals in the non‐tailored communication arm and 1.04 (95% CI = 0.93, 1.17) for individuals in the tailored communication arm compared with individuals in the SoC control arm.

### 3.3 Sensitivity analyses

Approximately 111 and 2804 individuals in the non‐tailored and tailored communication arms, respectively, resided in a household where no program communication was received during the program period and were excluded from the sensitivity analysis. Among the remaining sample, the unadjusted odds of receiving an influenza vaccination during the program period were 1.02 for individuals who received a non‐tailored communication (95% CI = 0.91, 1.14) and 0.91 (95% CI = 0.81, 1.02) among individuals who received a tailored communication, compared with individuals in the control arm.

### 3.4 Participant survey

A total of 6209 participants aged ≥18 years responded to the MyChart survey. Demographic characteristics of survey respondents are reported in Table 2. Overall, 79.1% (*n* = 4908) self‐reported as vaccinated during the 2020–2021 season; of these, 91.3% (*n* = 4483) had a recorded vaccination in the Reliant EHR system. Approximately 42.3% of respondents who self‐reported as unvaccinated recorded not receiving a recommendation for influenza vaccination from their healthcare provider, in contrast to only 15.8% who self‐reported as vaccinated (*p* < 0.001, Table 3). Among self‐reported unvaccinated respondents, 42.1% cited a lack of concern of their risk of influenza infection as the reason for not getting a vaccination. Self‐reported vaccinated respondents were more likely to indicate that protecting others around them had a major effect on their decision to get vaccinated compared with self‐reported unvaccinated respondents (39.9% vs. 10.4%, *p* < 0.001) and were more aware that getting vaccinated could protect themselves and higher risk individuals around them (92.6% vs. 82.3%, *p* < 0.001). Respondents who self‐reported as vaccinated more frequently indicated that COVID‐19 had a major impact on their decision to get vaccinated, compared with non‐vaccinated respondents (28.9% vs. 14.3%, *p* < 0.001), whereas unvaccinated respondents more frequently indicated it had no impact (51.6% vs. 34.2%, *p* < 0.001).

**TABLE 2:** Demographic characteristics of individuals who responded to the patient survey

	Total (*n* = 6209)	Control (*n* = 2163)	Non‐tailored communication (*n* = 2011)	Tailored communication (*n* = 2035)	*p* value
Age group, *n* (%)					0.155
<18 years	0 (0.0)	0 (0.0)	0 (0.0)	0 (0.0)	
18–49 years	3015 (48.6)	1050 (48.5)	954 (47.4)	1011 (49.7)	
50–64 years	2048 (33.0)	733 (33.9)	648 (32.2)	667 (32.8)	
≥65 years	1146 (18.5)	380 (17.6)	409 (20.3)	357 (17.5)	
Sex, *n* (%)					0.295
Female	4020 (64.7)	1414 (65.4)	1314 (65.3)	1292 (63.5)	
Male	2189 (35.3)	749 (34.6)	697 (34.7)	743 (36.5)	
Nonbinary/missing	0 (0.0)	0 (0.0)	0 (0.0)	0 (0.0)	
Race/ethnicity, *n* (%)					0.062
White	4866 (78.4)	1677 (77.5)	1600 (79.6)	1589 (78.1)	
Black	85 (1.4)	35 (1.6)	18 (0.9)	32 (1.6)	
Hispanic	137 (2.2)	43 (2.0)	43 (2.1)	51 (2.5)	
Asian/Pacific Islander	254 (4.1)	115 (5.3)	67 (3.3)	72 (3.5)	
Native American	24 (0.4)	7 (0.3)	9 (0.5)	8 (0.4)	
Unknown	825 (13.3)	279 (12.9)	267 (13.3)	279 (13.7)	
Missing	18 (0.3)	7 (0.3)	7 (0.4)	4 (0.2)	
Health plan type, *n* (%)					0.754
Commercial	4664 (75.1)	1629 (75.3)	1492 (74.2)	1543 (75.8)	
Medicare	1023 (16.5)	352 (16.3)	353 (17.6)	318 (15.6)	
Medicaid	366 (5.9)	121 (5.6)	121 (6.0)	124 (6.1)	
Medicare/Medicaid dual eligible	11 (0.2)	3 (0.1)	4 (0.2)	4 (0.2)	
Other/unknown	145 (2.3)	58 (2.7)	41 (2.0)	46 (2.3)	
Influenza risk status, *n* (%)					0.647
Average risk	2953 (47.6)	1036 (47.9)	939 (46.7)	978 (48.1)	
High risk	3256 (52.4)	1127 (52.1)	1072 (53.3)	1057 (51.9)	
Vaccinated during influenza season (8/1/2020 to 3/31/2021)^a^					0.609
Yes	4603 (74.1)	1615 (74.7)	1475 (73.4)	1513 (74.4)	
No	1606 (25.9)	548 (25.3)	536 (26.7)	522 (25.7)	

^a^ Based on data from electronic health records.

**TABLE 3:** Patient survey responses among individuals who completed the survey by self‐reported vaccination status

	Total (*n* = 6164)	Vaccinated (*n* = 4908)	Not vaccinated (*n* = 1256)	*p* value
	*n* (%)	*n* (%)	*n* (%)	*n* (%)
Q1. Has a healthcare provider recommended that you get a flu vaccine this year?				
Yes	4686 (76.0)	3988 (81.3)	698 (55.6)	<0.001
No	1308 (21.2)	777 (15.8)	531 (42.3)	<0.001
No response	170 (2.8)	143 (2.9)	27 (2.2)	0.141
Q2. Did you receive a flu vaccine this flu season (on or after August 1, 2020)?				
Yes	4908 (79.6)	4908 (100.0)	0 (0.0)	‐
No	1256 (20.4)	0 (0.0)	1256 (100.0)	‐
Q3. If you answered No to Question 2, please tell us why you did not get your flu vaccine this season (select all that apply).				
Valid *n*	1256	1256	1256	
No concerned about my risk of getting the flu; do not need one	529 (42.1)	‐	529 (42.1)	‐
I am allergic (egg or other vaccine allergy)	43 (3.4)	‐	43 (3.4)	‐
I do not have time to schedule a flu shot	71 (5.7)	‐	71 (5.7)	‐
I cannot afford the flu vaccine	4 (0.3)	‐	4 (0.3)	‐
The flu vaccine does not offer health benefits/does not work	82 (6.5)	‐	82 (6.5)	‐
I am avoiding clinics/pharmacies because of the COVID‐19 pandemic	151 (12.0)	‐	151 (12.0)	‐
No reason/other reason	473 (37.7)	‐	473 (37.7)	‐
N/A—I received a flu vaccine this year	12 (1.0)	‐	12 (1.0)	‐
Q4. If you answered Yes to Question 2, please tell us what prompted you to get your flu vaccine (select all that apply).				
Valid *n*	4907	4907	4907	
Concerned about my risk of getting the flu	1554 (31.7)	1544 (31.7)	‐	‐
I always get one	3254 (66.3)	3254 (66.3)	‐	‐
Communication/reminder from a healthcare provider	163 (3.3)	163 (3.3)	‐	‐
Suggested by family/friends	287 (5.9)	287 (5.9)	‐	‐
Recommended by a healthcare provider	955 (19.5)	955 (19.5)	‐	‐
No reason/other reason	319 (6.5)	319 (6.5)	‐	‐
N/A—I did not receive a flu vaccine this year	23 (0.5)	23 (0.5)	‐	‐
Q5. On a scale of 1–5, how much did the role of protecting those around you (loved ones, friends, household members, etc.) affect your decision to receive or not receive a flu vaccine this season?				
No affect	1205 (19.6)	648 (13.2)	557 (44.4)	<0.001
Minor affect	322 (5.2)	261 (5.3)	61 (4.9)	0.513
Neutral	1161 (18.8)	781 (15.9)	380 (30.3)	<0.001
Moderate affect	1247 (20.2)	1157 (23.6)	90 (7.2)	<0.001
Major affect	2090 (33.9)	1960 (39.9)	130 (10.4)	<0.001
No response	139 (2.3)	101 (2.1)	38 (3.0)	0.039
Q6. Were you aware that getting a flu vaccine can help protect you and any higher risk individuals (e.g., older adults and those with chronic health conditions) around you?				
Yes	5578 (90.5)	4544 (92.6)	1034 (82.3)	<0.001
No	449 (7.3)	267 (5.4)	182 (14.5)	<0.001
No response	137 (2.2)	97 (2.0)	40 (3.2)	0.010
Q7. On a scale of 1–5, how much did the ongoing COVID‐19 pandemic affect your decision to receive or not receive a flu vaccine this season?				
No affect	2325 (37.7)	1677 (34.2)	648 (51.6)	<0.001
Minor affect	347 (5.6)	284 (5.8)	63 (5.0)	0.288
Neutral	890 (14.4)	683 (13.9)	207 (16.5)	0.022
Moderate affect	960 (15.6)	813 (16.6)	147 (11.7)	<0.001
Major affect	1597 (25.9)	1418 (28.9)	179 (14.3)	<0.001
No response	45 (0.7)	33 (0.7)	12 (1.0)	0.282

*Note*: Excluded 45 respondents who did not self‐report vaccination status (i.e., did not answer Question 2).

## 4 DISCUSSION

The objective of this study was to develop and evaluate a novel household‐based recall–reminder intervention to increase late‐season influenza vaccine uptake, leveraging pre‐existing technological systems within care delivery organizations. During the program period, influenza vaccination rates among unvaccinated individuals in eligible households increased by only 3.3%, highlighting the degree to which vaccine uptake tapers off in the latter part of the influenza season. There were no significant differences observed in uptake between the intervention arms relative to the SoC control arm, indicating that the tailored and non‐tailored communications did not have an effect.

It is important, however, to interpret these results within the context in which this study was conducted. The concurrent COVID‐19 pandemic provided substantial challenges hindering the effectiveness of the intervention. During the nearly 2‐month delay between randomization and program start, 11.1% of unvaccinated individuals in the randomized population received an influenza vaccine, rendering more than 25% of households ineligible. Furthermore, the program now targeted the latter portion of the already late season (i.e., January–February), thus restricting the sample to an even more vaccine‐hesitant subset of the patient population than originally anticipated. Also unanticipated was the availability of COVID‐19 vaccines, which Reliant began offering in February 2021, coinciding with the study period. Outreach communications were sometimes misinterpreted as COVID‐19‐related, despite efforts to clearly emphasize influenza vaccination, especially with newly competing interests for COVID‐19 vaccination appointments. Increasing occurrences of misinterpretations, in combination with resource constraints, led to early termination of study communications and thus, not all patients randomized to receive an IVR communication were contacted.

Despite these results, we believe there are merits inherent to this approach that warrant further exploration. Following the COVID‐19 pandemic, healthcare services have increasingly become remote, and there will likely be a continued need for effective outreach programs to increase patient engagement. The sentiments of protecting those around you will also likely last beyond the first years of the pandemic, such that an intervention like this will still resonate well among household members in the coming years. Additionally, this type of program is inherently flexible, so messages can be tailored based upon the circumstances of the year in which they are delivered. Above all, this household‐based intervention approach is efficient to scale up across healthcare systems and can be adapted to other vaccination programs, including COVID‐19 vaccines, given the flexible design and use of existing clinic infrastructure.

Results from the patient survey revealed additional insights that are worth highlighting. Our findings reinforce the sentiment that protecting household members and high‐risk individuals is a key behavioral driver that plays an important role in the decision of whether to receive the influenza vaccine. While most self‐reported vaccinated participants were aware of their own risk of influenza infection and that getting vaccinated can protect their contacts, there remains an important knowledge gap among those unvaccinated, which may be addressed through continued patient support and education from providers. Additionally, less than half of self‐reported unvaccinated survey respondents received a recommendation for influenza vaccination from their healthcare provider, which is known to be strong predictor of influenza vaccine uptake.^13^, ^14^, ^15^, ^16^, ^17^ Because the COVID‐19 pandemic has had a substantial effect on perceptions of vaccination and hesitancy,^18^, ^19^, ^20^, ^21^, ^22^ it remains to be seen if and how these attitude changes may persist in the coming years. Given the gravity of the COVID‐19 pandemic, it is plausible that the sentiment of protecting contacts may continue to be an important behavioral driver in one's decision to be vaccinated.

Several study design limitations arose during program implementation. Logistically, identifying households based on EHR data was complicated and resource intensive, requiring manual normalization and linkage of several fields, thus jeopardizing the accuracy of these identifiers. Implementing household identifier fields into EHR systems could easily mitigate this challenge in the future. As only patients clinically active in Reliant's system were enrolled, households may have had members outside of Reliant's system; thus, communications may not have reached all household members. Implementation of the definition of high‐risk groups for influenza‐ or COVID‐19‐related illness was logistically challenging using the available EHR system; by instead using the current problems list, some households may have been misclassified. Further, conditions were less likely to be captured from an EHR during 2020 given the infrequency of routine provider visits during the pandemic. Lastly, the higher rates of self‐reported versus EHR‐recorded influenza vaccination observed among patients who responded to the patient survey suggest that vaccination history data collected in the Reliant EHR system may be incomplete. However, comparing vaccination rates among the full sample of randomized individuals (~51.7%) to that of the estimated national average (52.6%)^23^ suggests that the magnitude of this issue is likely trivial.

Consistent with data reported elsewhere,^10^ we observed vaccination rates tapering off after January, with few people vaccinated during the program period. In the United States, most influenza cases occur from late December through March, reinforcing the importance of promoting influenza vaccination even after the typical campaign period (September–November) ends. Despite the pandemic‐related challenges in the operationalization of this pilot, we believe the household‐based approach to improving vaccination rates remains promising and worthy of further exploration, incorporating insights gained and lessons learned from this study and others recently published.^24^

## CONFLICT OF INTEREST

MML and MG are full‐time employees of Sanofi, a company that makes influenza vaccines, and may hold shares and/or stock options in the company. NP, TK, and AS are employees of Optum Life Sciences. MP was an employee of Optum Life Sciences at the time the study was conducted and is currently employed by the Henry M. Jackson Foundation in support of the US Military HIV Research Program (MHRP) and Emerging Infectious Diseases Branch (EIDB). LF, MC, CL, and DS are employees of Reliant Medical Group.

## AUTHOR CONTRIBUTIONS

LF was involved in conceptualization of the study and reviewing the manuscript. MML participated in methodology, conceptualization, and reviewing and editing the manuscript. NP participated in conceptualization of the study, project administration, and reviewing and editing the manuscript. TK was involved in conceptualization and supervision of the study and reviewing and editing the manuscript. MG participated in conceptualization and supervision of the study and reviewing the manuscript. MC participated in conceptualization of the study, project administration, and reviewing the manuscript. CL was involved in project administration and reviewing the manuscript. DS participated in data curation, validation, and reviewing the manuscript. TB participated in formal analysis and validation and reviewing and editing the manuscript. AS was involved in methodology, formal analysis, and reviewing and editing the manuscript. MP participated in methodology, formal analysis and validation, and reviewing and editing the manuscript.

## Supporting information


**Table S1.** Conditions included in the definition for high‐risk of influenza complications

**Table S2.** Patient communications sent via interactive voice response or through MyChart

**Table S3.** Randomization characteristics of randomized households and eligible households^1^

**Table S4.** Demographic characteristics of individuals from all randomized households

**Figure S1.** Randomization scheme of the full study population^1^
^1^In each flow diagram box, four sets of numbers are presented. The first are counts of the unique number of households in the full sample (as randomized) and the second counts are the number of eligible households excluding households that became fully vaccinated prior to the start of the program. The third count is the number of unique individuals in the randomized sample and the fourth counts are the number of eligible individuals after excluding those who were vaccinated prior to the start of the program.
IVR, interactive voice response

## Data Availability

The data contained in our database contains proprietary elements owned by Optum and, therefore, cannot be broadly disclosed or made publicly available at this time. The disclosure of this data to third‐party clients assumes certain data security and privacy protocols are in place and that the third‐party client has executed our standard license agreement which includes restrictive covenants governing the use of the data.
