# Supplementary material for: A novel household‐based patient outreach pilot program to boost late‐season influenza vaccination rates during the COVID‐19 pandemic
Source: Influenza Other Respir Viruses. 2022 Sep 13;16(6):1141–50. doi: 10.1111/irv.13041 (PMC9530505; doi:10.1111/irv.13041)
Supplement: Supplementary file 1 — Table S1. Conditions included in the definition for high‐risk of influenza complications [file IRV-16-1141-s005.docx]

Supplementary Table 1. Conditions included in the definition for high-risk of influenza complications

| Chronic obstructive pulmonary disease |
| --- |
| Cancer |
| Chronic kidney disease |
| Type 1 or 2 diabetes mellitus |
| Obesity |
| Immunocompromised (ie, solid organ transplant) |
| Heart failure, coronary artery disease, or cardiomyopathies |
| Sickle cell disease |
| Stroke |
| Asthma |
| Cystic Fibrosis |
| Weakened immune system from HIV/AIDS, leukemia/lymphoma, or taking certain medications such as chemotherapy, radiation therapy, chronic corticosteroids, or other immunosuppressive medications |
| Liver Disease |
| Neurologic and neurodevelopment conditions including dementia |
| Pregnant and/or delivered in the past 2 weeks |
| Hypertension |
| Metabolic disorders |
| Pulmonary fibrosis |
| Age <19 and taking long-term aspirin or salicylate containing medications |
| Thalassemia |
