# Supplementary material for: A novel household‐based patient outreach pilot program to boost late‐season influenza vaccination rates during the COVID‐19 pandemic
Source: Influenza Other Respir Viruses. 2022 Sep 13;16(6):1141–50. doi: 10.1111/irv.13041 (PMC9530505; doi:10.1111/irv.13041)
Supplement: Supplementary file 2 — Table S2. Patient communications sent via interactive voice response or through MyChart [file IRV-16-1141-s002.docx]

Supplementary Table 2. Patient communications sent via interactive voice response or through MyChart

| **Non-tailored Communication** | | |
| --- | --- | --- |
| Interactive voice response (IVR, automated call) | Hello, this is Reliant Medical Group. This year, getting a seasonal flu vaccine is more important than ever. Seasonal flu vaccines are the best way to protect yourself, your loved ones, and others in the community from the flu. If you or any other Reliant patient in your home has not yet received a flu vaccine, it’s not too late.  If you have an upcoming appointment in primary care, or even in most specialty departments, you can get your flu shot then. If not, please contact us at our dedicated seasonal flu vaccine line xxx-xxx-xxxx between the hours of 8 am and 4:30 pm Monday through Friday. We can schedule an appointment for any Reliant patient in your home that hasn’t yet received their flu vaccine. Do not call your primary physician’s office to make this appointment.  Flu vaccines are available at all Reliant primary care offices, so you can schedule an appointment at the office most convenient to you. |  |
| MyChart message | **Headline: Has everyone in your home received a seasonal flu vaccine?**  This year, getting a seasonal flu vaccine is more important than ever. Seasonal flu vaccines are the best way to protect yourself, your loved ones, and others in the community from the flu. If you or any other Reliant patient in your home has not yet received a flu vaccine, it’s not too late.  If you have an upcoming appointment in primary care, or even in most specialty departments, you can get your flu shot then. If not, we’ve made scheduling an appointment for a flu vaccine easy. You can call our dedicated seasonal flu vaccine line during the week at xxx-xxx-xxxx between 8:00am-4:30pm. Flu vaccines are available at all Reliant primary care offices, so you can schedule an appointment at the office most convenient for you. Do not call your primary physician’s office to make this appointment.  If you or any other Reliant patient in your home has not yet received a flu vaccine, make an appointment today! |  |
| **Tailored Communication** | |  |
| Interactive voice response (IVR, automated call) | Hello, this is Reliant Medical Group. This year, getting a seasonal flu vaccine is more important than ever – especially for people over 65 years old or for those with chronic medical conditions, like heart and lung disease, diabetes, asthma, and cancer. Seasonal flu vaccination has been shown to decrease the risk of being hospitalized or experiencing life-threatening complications from the flu, and is the best way to protect you, your loved ones, and the community from the flu. If you or any other Reliant patient in your home has not yet received a flu vaccine, it’s not too late.  If you have an upcoming appointment in primary care, or even in most specialty departments, you can get your flu shot then. If not, please contact Reliant directly on our dedicated seasonal influenza vaccination phone line xxx-xxx-xxxx between the hours of 8 am and 4:30 pm Monday through Friday as soon as possible to schedule an appointment for any Reliant patient in your home that hasn’t yet received their flu vaccine. Do not call your primary physician’s office to make this appointment.  Flu vaccines are available at all Reliant primary care offices, so you can schedule an appointment at the office most convenient for you. |  |
| MyChart message | **Headline: Has everyone in your home received a seasonal flu vaccine?**  This year, getting a seasonal flu vaccine is more important than ever – especially for people over 65 years old or for those with chronic medical conditions, like heart and lung disease, diabetes, asthma, and cancer. Even if you do not have a high-risk condition, getting a flu shot is still important to help protect others around you who are at higher risk.  Seasonal flu vaccination has been shown to decrease the risk of being hospitalized or experiencing life-threatening complications from the flu, and is the best way to protect you, your loved ones, and the community from the flu. If you or any other Reliant patient in your home has not yet received a flu vaccine, it’s not too late.  If you have an upcoming appointment in primary care, or even in most specialty departments, you can get your flu shot then. If not, we’ve made scheduling an appointment for a flu vaccine easy. You can call our dedicated seasonal flu vaccine line during the week at xxx-xxx-xxxx between 8:00am-4:30pm. Flu vaccines are available at all Reliant primary care offices, so you can schedule an appointment at the office most convenient for you. Do not call your primary physician’s office to make this appointment.  If you or any other Reliant patient in your home has not yet received a flu vaccine, it’s not too late. Make an appointment today! |  |
