# Supplementary material for: A novel household‐based patient outreach pilot program to boost late‐season influenza vaccination rates during the COVID‐19 pandemic
Source: Influenza Other Respir Viruses. 2022 Sep 13;16(6):1141–50. doi: 10.1111/irv.13041 (PMC9530505; doi:10.1111/irv.13041)
Supplement: Supplementary file 3 — Table S3. Randomization characteristics of randomized households and eligible households1 [file IRV-16-1141-s004.docx]

Supplementary Table 3. Randomization characteristics of randomized households and eligible households^1^

|  | **Total**  **(n = 36,920)** | **Control**  **(n = 12,436)** | **Non-tailored communication (n = 11,991)** | **Tailored communication (n = 12,493)** | **p-value** |
| --- | --- | --- | --- | --- | --- |
| **Randomized Households** |  |  |  |  |  |
| Number of clinically active patients per household, mean (SD) | 2.6 (0.9) | 2.6 (0.9) | 2.6 (0.9) | 2.6 (0.9) | 0.002 |
| Reliant Medical Group clinic, n (%) |  |  |  |  | 0.287 |
| Auburn | 6,333 (17.2) | 2,128 (17.1) | 2,089 (17.4) | 2,116 (16.9) |  |
| Holden | 3,222 (8.7) | 1,130 (9.1) | 1,041 (8.7) | 1,051 (8.4) |  |
| Leominster | 3,984 (10.8) | 1,327 (10.7) | 1,284 (10.7) | 1,373 (11.0) |  |
| Milford | 2,309 (6.3) | 750 (6.0) | 778 (6.5) | 781 (6.3) |  |
| Neponset | 4,901 (13.3) | 1,625 (13.1) | 1,578 (13.2) | 1,698 (13.6) |  |
| Shrewsbury | 2,706 (7.3) | 886 (7.1) | 867 (7.2) | 953 (7.6) |  |
| SMG Framingham | 2,413 (6.5) | 819 (6.6) | 795 (6.6) | 799 (6.4) |  |
| SMG Southborough | 5,995 (16.2) | 2,003 (16.1) | 1,934 (16.1) | 2,058 (16.5) |  |
| Webster | 917 (2.5) | 310 (2.5) | 297 (2.5) | 310 (2.5) |  |
| Westboro Union Street | 3,892 (10.5) | 1,354 (10.9) | 1,263 (10.5) | 1,275 (10.2) |  |
| Worcester | 248 (0.7) | 104 (0.8) | 65 (0.5) | 79 (0.6) |  |
| Age Categories, n (%) |  |  |  |  |  |
| Households with individuals  <18 years | 16,899 (45.8) | 5,689 (45.8) | 5,471 (45.6) | 5,739 (45.9) | 0.885 |
| Households with individuals 18-  49 years | 25,551 (69.2) | 8,572 (68.9) | 8,294 (69.2) | 8,685 (69.5) | 0.598 |
| Households with individuals 50-  64 years | 13,524 (36.6) | 4,576 (36.8) | 4,376 (36.5) | 4,572 (36.6) | 0.883 |
| Households with individuals  ≥65 years | 6,326 (17.1) | 2,078 (16.7) | 2,150 (17.9) | 2,098 (16.8) | 0.019 |
| Households with individuals  <18 at high risk | 7,901 (21.4) | 2,650 (21.3) | 2,593 (21.6) | 2,658 (21.3) | 0.765 |
| Households with individuals 18-  49 years at high risk | 15,033 (40.7) | 5,008 (40.3) | 4,891 (40.8) | 5,134 (41.1) | 0.408 |
| Households with individuals 50-  64 years at high risk | 10,536 (28.5) | 3,525 (28.4) | 3,432 (28.6) | 3,579 (28.7) | 0.843 |
| Households with individuals  ≥65 years at high risk | 5,970 (16.2) | 1,966 (15.8) | 2,027 (16.9) | 1,977 (15.8) | 0.029 |
|  | **Total**  **(n = 27,658)** | **Control**  **(n = 9,282)** | **Non-tailored communication (n = 8,991)** | **Tailored communication (n = 9,385)** | **p-value** |
| **Eligible Households^1^** |  |  |  |  |  |
| Number of clinically active patients per household, mean (SD) | 2.6 (0.9) | 2.6 (0.9) | 2.6 (0.9) | 2.6 (1.0) | <0.001 |
| Reliant Medical Group clinic, n (%) |  |  |  |  | 0.346 |
| Auburn | 4,885 (17.7) | 1,621 (17.5) | 1,630 (18.1) | 1,634 (17.4) |  |
| Holden | 2,447 (8.9) | 849 (9.2) | 801 (8.9) | 797 (8.5) |  |
| Leominster | 3,249 (11.8) | 1,089 (11.7) | 1,034 (11.5) | 1,126 (12.0) |  |
| Milford | 1,962 (7.1) | 647 (7.0) | 649 (7.2) | 666 (7.1) |  |
| Shrewsbury | 2,005 (7.3) | 662 (7.1) | 635 (7.1) | 708 (7.5) |  |
| Framingham | 1,773 (6.4) | 584 (6.3) | 594 (6.6) | 595 (6.3) |  |
| Southborough | 3,979 (14.4) | 1,330 (14.3) | 1,300 (14.5) | 1,349 (14.4) |  |
| Webster | 774 (2.8) | 260 (2.8) | 252 (2.8) | 262 (2.8) |  |
| Westboro | 2,681 (9.7) | 948 (10.2) | 860 (9.6) | 873 (9.3) |  |
| Worcester | 3,716 (13.4) | 1,214 (13.1) | 1,187 (13.2) | 1,315 (14.0) |  |
| Worcester 2 | 187 (0.7) | 78 (0.8) | 49 (0.5) | 60 (0.6) |  |
| Age Categories, n (%) |  |  |  |  |  |
| Households with individuals  <18 years | 12,215 (44.2) | 4,101 (44.2) | 3,940 (43.8) | 4,174 (44.5) | 0.671 |
| Households with individuals 18-  49 years | 20,491 (74.1) | 6,855 (73.9) | 6,670 (74.2) | 6,966 (74.2) | 0.817 |
| Households with individuals 50-  64 years | 10,606 (38.4) | 3,562 (38.4) | 3,427 (38.1) | 3,617 (38.5) | 0.838 |
| Households with individuals  ≥65 years | 4,185 (15.1) | 1,356 (14.6) | 1,434 (16.0) | 1,395 (14.9) | 0.028 |
| Households with individuals  <18 at high risk | 5,903 (21.3) | 1,963 (21.2) | 1,945 (21.6) | 1,995 (21.3) | 0.705 |
| Households with individuals 18-  49 years at high risk | 12,213 (44.2) | 4,036 (43.5) | 3,995 (44.4) | 4,182 (44.6) | 0.271 |
| Households with individuals 50-  64 years at high risk | 8,242 (29.8) | 2,731 (29.4) | 2,690 (29.9) | 2,821 (30.1) | 0.609 |
| Households with individuals  ≥65 years at high risk | 3,925 (14.2) | 1,272 (13.7) | 1,350 (15.0) | 1,303 (13.9) | 0.023 |

^1^Excludes households fully vaccinated before program start (8/1/2020–1/12/2021)
