# Supplementary material for: A novel household‐based patient outreach pilot program to boost late‐season influenza vaccination rates during the COVID‐19 pandemic
Source: Influenza Other Respir Viruses. 2022 Sep 13;16(6):1141–50. doi: 10.1111/irv.13041 (PMC9530505; doi:10.1111/irv.13041)
Supplement: Supplementary file 4 — Table S4. Demographic characteristics of individuals from all randomized households [file IRV-16-1141-s003.docx]

Supplementary Table 4. Demographic characteristics of individuals from all randomized households

|  | **Total**  **(n = 94,747)** | **Control**  **(n = 31,803)** | **Non-tailored communication (n = 30,599)** | **Tailored communication (n =** **32,345)** | **p-value** |
| --- | --- | --- | --- | --- | --- |
| Age group, n (%) |  |  |  |  | 0.068 |
| <18 years | 27,938 (29.5) | 9,357 (29.4) | 8,962 (29.3) | 9,619 (29.7) |  |
| 18-49 years | 38,468 (40.6) | 12,902 (40.6) | 12,372 (40.4) | 13,194 (40.8) |  |
| 50-64 years | 18,802 (19.8) | 6,409 (20.2) | 6,025 (19.7) | 6,368 (19.7) |  |
| ≥65 years | 9,539 (10.1) | 3,135 (9.9) | 3,240 (10.6) | 3,164 (9.8) |  |
| Sex, n (%) |  |  |  |  | 0.834 |
| Female | 50,188 (53.0) | 16,874 (53.1) | 16,246 (53.1) | 17,068 (52.8) |  |
| Male | 44,554 (47.0) | 14,928 (46.9) | 14,351 (46.9) | 15,275 (47.2) |  |
| Nonbinary/missing | 5 (0.01) | 1 (0.0) | 2 (0.01) | 2 (0.01) |  |
| Race/ethnicity, n (%) |  |  |  |  | 0.003 |
| White | 61,451 (64.9) | 20,469 (64.4) | 20,024 (65.4) | 20,958 (64.8) |  |
| Black | 3,223 (3.4) | 1,066 (3.4) | 1,022 (3.3) | 1,135 (3.5) |  |
| Hispanic | 4,998 (5.3) | 1,643 (5.2) | 1,582 (5.2) | 1,773 (5.5) |  |
| Asian/Pacific Islander | 5,631 (5.9) | 2,100 (6.6) | 1,683 (5.5) | 1,848 (5.7) |  |
| Native American | 285 (0.3) | 99 (0.3) | 90 (0.3) | 96 (0.3) |  |
| Unknown | 18,415 (19.4) | 6,186 (19.5) | 5,979 (19.5) | 6,250 (19.3) |  |
| Missing | 744 (0.8) | 240 (0.8) | 219 (0.7) | 285 (0.9) |  |
| MyChart active status, n (%) | 64,267 (67.8) | 21,555 (67.8) | 20,800 (68.0) | 21,912 (67.7) | 0.871 |
| Health plan type, n (%) |  |  |  |  | 0.012 |
| Commercial | 62,949 (66.4) | 21,301 (67.0) | 20,214 (66.1) | 21,434 (66.3) |  |
| Medicare | 8,605 (9.1) | 2,822 (8.9) | 2,938 (9.6) | 2,845 (8.8) |  |
| Medicaid | 14,541 (15.4) | 4,739 (14.9) | 4,646 (15.2) | 5,156 (15.9) |  |
| Medicare/Medicaid dual  eligible | 99 (0.1) | 33 (0.1) | 38 (0.1) | 28 (0.1) |  |
| Other/unknown | 8,553 (9.0) | 2,908 (9.1) | 2,763 (9.0) | 2,882 (8.9) |  |
| Influenza risk status, n (%) |  |  |  |  | 0.263 |
| Average risk | 60,909 (64.3) | 20,507 (64.5) | 19,542 (63.9) | 20,860 (64.5) |  |
| High risk | 33,838 (35.7) | 11,296 (35.5) | 11,057 (36.1) | 11,485 (35.5) |  |
| Vaccinated prior to program start (8/1/2020 to 1/12/2021) |  |  |  |  | 0.186 |
| Yes | 46,564 (49.2) | 15,730 (49.5) | 15,102 (49.4) | 15,732 (48.6) |  |
| No | 48,183 (50.9) | 16,073 (50.5) | 15,497 (50.7) | 16,613 (51.4) |  |
| Vaccinated during influenza season (8/1/2020 to 3/31/2021) |  |  |  |  | 0.301 |
| Yes | 48,953 (51.7) | 16,511 (51.9) | 15,869 (51.9) | 16,573 (51.2) |  |
| No | 45,794 (48.3) | 15,292 (48.1) | 14,730 (48.1) | 15,772 (48.8) |  |
