# Supplementary figures and images for: A novel household‐based patient outreach pilot program to boost late‐season influenza vaccination rates during the COVID‐19 pandemic
Source: Influenza Other Respir Viruses. 2022 Sep 13;16(6):1141–50. doi: 10.1111/irv.13041 (PMC9530505; doi:10.1111/irv.13041)

Supplementary Figure 1


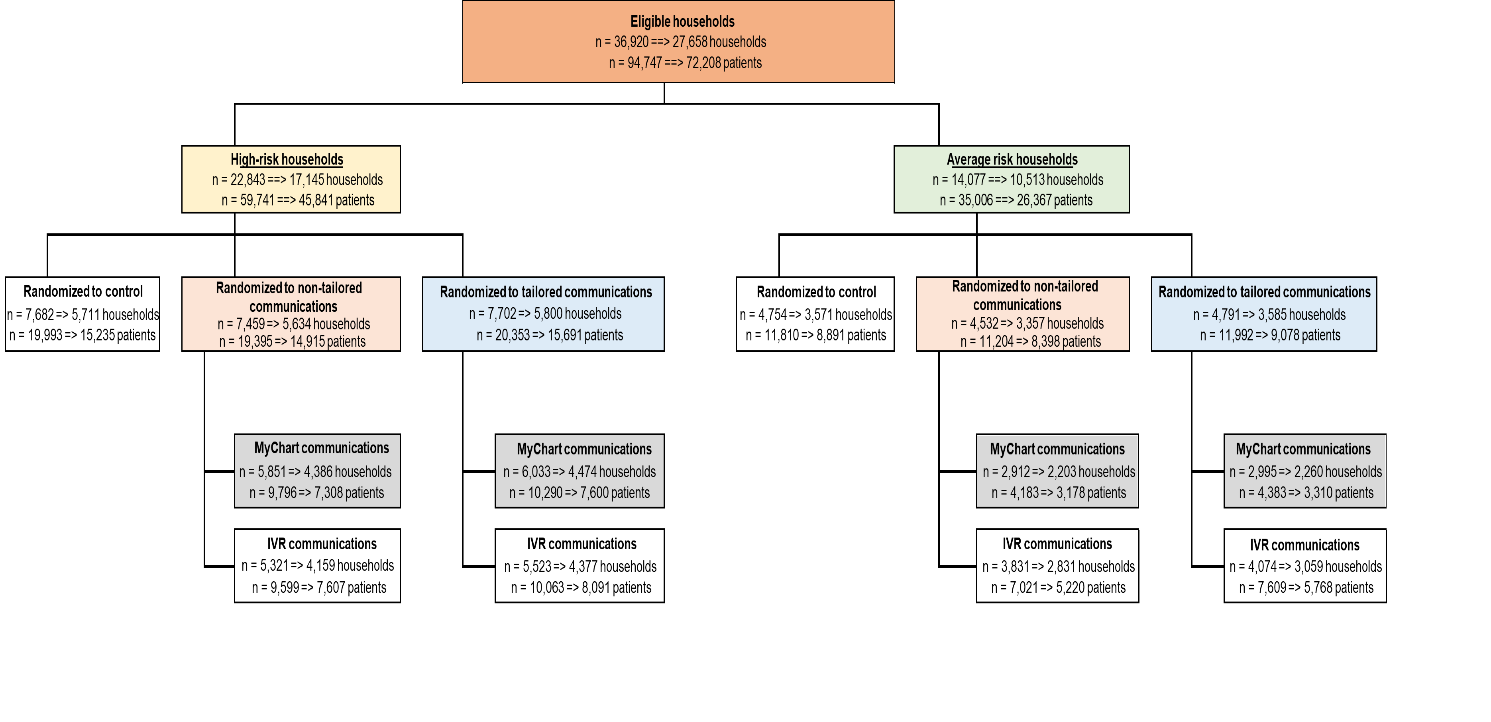

Supplement: Supplementary file 5 — Figure S1. Randomization scheme of the full study population1 1In each flow diagram box, four sets of numbers are presented. The first are counts of the unique number of households in the full sample (as randomized) and the second counts are the number of eligible households excluding households that became fully vaccinated prior to the start of the program. The third count is the number of unique individuals in the randomized sample and the fourth counts are the number of eligible individuals after excluding those who were vaccinated prior to the start of the program. IVR, interactive voice response [file IRV-16-1141-s001.docx]
